# 1-(4-Methyl­phen­yl)-2-(phenyl­sulfon­yl)ethanone

**DOI:** 10.1107/S160053681200966X

**Published:** 2012-03-10

**Authors:** Hatem A. Abdel-Aziz, Khalid A. Al-Rashood, Hazem A. Ghabbour, Hoong-Kun Fun, Tze Shyang Chia

**Affiliations:** a Department of Pharmaceutical Chemistry, College of Pharmacy, King Saud University, PO Box 2457, Riyadh 11451, Saudi Arabia; b X-ray Crystallography Unit, School of Physics, Universiti Sains Malaysia, 11800 USM, Penang, Malaysia

## Abstract

In the title compound, C_15_H_14_O_3_S, the benzene and phenyl rings make a dihedral angle of 33.56 (16)°. In the crystal, mol­ecules are linked by C—H⋯O hydrogen bonds into a layer parallel to the *ab* plane.

## Related literature

For background to the chemistry of sulfones, see: Xiang *et al.* (2007 ▶); Abdel-Aziz & Mekawey (2009 ▶); Abdel-Aziz *et al.* (2010 ▶). For a related structure, see: Abdel-Aziz *et al.* (2011 ▶). For reference bond lengths, see: Allen *et al.* (1987 ▶).
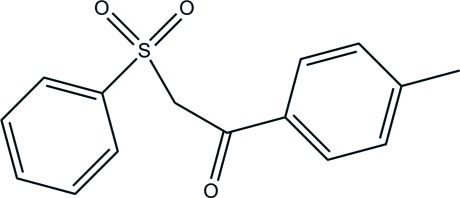


## Experimental

### 

#### Crystal data

C_15_H_14_O_3_S
*M* *_r_* = 274.32
Orthorhombic, 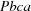

*a* = 11.5555 (12) Å
*b* = 10.1981 (11) Å
*c* = 22.843 (2) Å
*V* = 2692.0 (5) Å^3^
*Z* = 8
Cu *K*α radiation
μ = 2.15 mm^−1^
*T* = 296 K
0.55 × 0.12 × 0.04 mm

#### Data collection

Bruker SMART APEXII CCD area-detector diffractometer
Absorption correction: multi-scan (*SADABS*; Bruker, 2009 ▶) *T*_min_ = 0.386, *T*_max_ = 0.919
10168 measured reflections
2487 independent reflections
1446 reflections with *I* > 2σ(*I*)
*R*_int_ = 0.068

#### Refinement

*R*[*F* ^2^ > 2σ(*F* ^2^)] = 0.045
*wR*(*F* ^2^) = 0.124
*S* = 0.89
2487 reflections
174 parameters
H-atom parameters constrained
Δρ_max_ = 0.19 e Å^−3^
Δρ_min_ = −0.25 e Å^−3^

### 

Data collection: *APEX2* (Bruker, 2009 ▶); cell refinement: *SAINT* (Bruker, 2009 ▶); data reduction: *SAINT*; program(s) used to solve structure: *SHELXTL* (Sheldrick, 2008 ▶); program(s) used to refine structure: *SHELXTL*; molecular graphics: *SHELXTL*; software used to prepare material for publication: *SHELXTL* and *PLATON* (Spek, 2009 ▶).

## Supplementary Material

Crystal structure: contains datablock(s) global, I. DOI: 10.1107/S160053681200966X/is5087sup1.cif

Structure factors: contains datablock(s) I. DOI: 10.1107/S160053681200966X/is5087Isup2.hkl

Supplementary material file. DOI: 10.1107/S160053681200966X/is5087Isup3.cml

Additional supplementary materials:  crystallographic information; 3D view; checkCIF report

## Figures and Tables

**Table 1:** Hydrogen-bond geometry (Å, °)

*D*—H⋯*A*	*D*—H	H⋯*A*	*D*⋯*A*	*D*—H⋯*A*
C7—H7*B*⋯O1^i^	0.97	2.42	3.366 (3)	164
C10—H10*A*⋯O3^ii^	0.93	2.48	3.342 (4)	154

Symmetry codes: (i) 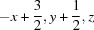; (ii) 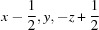.

**Fig. 1.**
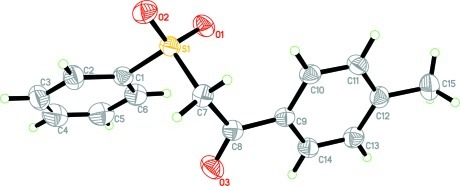
The molecular structure of the title compound with atom labels and 30% probability displacement ellipsoids.

**Fig. 2.**
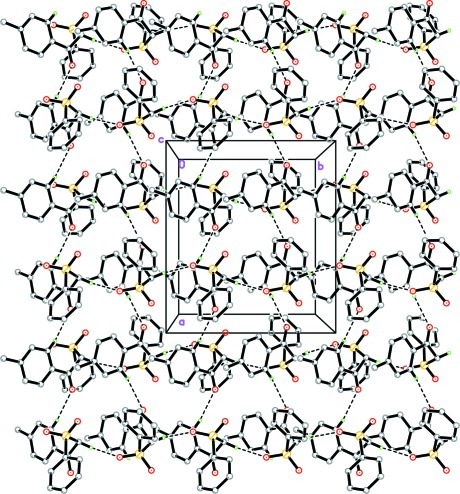
The crystal packing of the title compound. The dashed lines represent the hydrogen bonds. For clarity sake, hydrogen atoms not involved in hydrogen bonding have been omitted.

## supplementary crystallographic information

### Comment

In the title compound (Fig. 1), the two aromatic rings (C1–C6 and C9–C14) form a dihedral angle of 33.56 (16)° with each other. Bond lengths (Allen *et al.*, 1987) and angles are within normal ranges and are comparable to the related structure (Abdel-Aziz *et al.*, 2011). In the crystal packing (Fig. 2), the molecules are linked by intermolecular C7—H7B···O1 and C10—H10A···O3 hydrogen bonds (Table 1) into two-dimensional networks parallel to the *ab* plane.

### Experimental

The title compound was prepared according to the reported method (Xiang *et al.*, 2007). Single crystals of the title compound were obtained by slow evaporation from an ethanol solution at room temperature.

### Refinement

All H atoms were positioned geometrically (C—H = 0.93, 0.96 and 0.97 Å) and refined using a riding model, with *U*_iso_(H) = 1.2 and 1.5*U*_eq_(C).

### Crystal data

**Table:**

C_15_H_14_O_3_S	*F*(000) = 1152
*M**_r_* = 274.32	*D*_x_ = 1.354 Mg m^−^^3^
Orthorhombic, *P**b**c**a*	Cu *K*α radiation, λ = 1.54178 Å
Hall symbol: -P 2ac 2ab	Cell parameters from 484 reflections
*a* = 11.5555 (12) Å	θ = 3.9–33.7°
*b* = 10.1981 (11) Å	µ = 2.15 mm^−^^1^
*c* = 22.843 (2) Å	*T* = 296 K
*V* = 2692.0 (5) Å^3^	Needle, colourless
*Z* = 8	0.55 × 0.12 × 0.04 mm

### Data collection

**Table:**

Bruker SMART APEXII CCD area-detector diffractometer	2487 independent reflections
Radiation source: fine-focus sealed tube	1446 reflections with *I* > 2σ(*I*)
Graphite monochromator	*R*_int_ = 0.068
φ and ω scans	θ_max_ = 70.0°, θ_min_ = 3.9°
Absorption correction: multi-scan (*SADABS*; Bruker, 2009)	*h* = −11→14
*T*_min_ = 0.386, *T*_max_ = 0.919	*k* = −9→11
10168 measured reflections	*l* = −27→17

### Refinement

**Table:**

Refinement on *F*^2^	Secondary atom site location: difference Fourier map
Least-squares matrix: full	Hydrogen site location: inferred from neighbouring sites
*R*[*F*^2^ > 2σ(*F*^2^)] = 0.045	H-atom parameters constrained
*wR*(*F*^2^) = 0.124	*w* = 1/[σ^2^(*F*_o_^2^) + (0.0623*P*)^2^] where *P* = (*F*_o_^2^ + 2*F*_c_^2^)/3
*S* = 0.89	(Δ/σ)_max_ < 0.001
2487 reflections	Δρ_max_ = 0.19 e Å^−^^3^
174 parameters	Δρ_min_ = −0.25 e Å^−^^3^
0 restraints	Extinction correction: *SHELXTL* (Sheldrick, 2008), Fc^*^=kFc[1+0.001xFc^2^λ^3^/sin(2θ)]^-1/4^
Primary atom site location: structure-invariant direct methods	Extinction coefficient: 0.00131 (15)

### Special details

**Table:**

Geometry. All e.s.d.'s (except the e.s.d. in the dihedral angle between two l.s. planes) are estimated using the full covariance matrix. The cell e.s.d.'s are taken into account individually in the estimation of e.s.d.'s in distances, angles and torsion angles; correlations between e.s.d.'s in cell parameters are only used when they are defined by crystal symmetry. An approximate (isotropic) treatment of cell e.s.d.'s is used for estimating e.s.d.'s involving l.s. planes.
Refinement. Refinement of *F*^2^ against ALL reflections. The weighted *R*-factor *wR* and goodness of fit *S* are based on *F*^2^, conventional *R*-factors *R* are based on *F*, with *F* set to zero for negative *F*^2^. The threshold expression of *F*^2^ > σ(*F*^2^) is used only for calculating *R*-factors(gt) *etc*. and is not relevant to the choice of reflections for refinement. *R*-factors based on *F*^2^ are statistically about twice as large as those based on *F*, and *R*- factors based on ALL data will be even larger.

### Fractional atomic coordinates and isotropic or equivalent isotropic displacement parameters (Å^2^)

**Table:**

	*x*	*y*	*z*	*U*_iso_*/*U*_eq_	
S1	0.69612 (6)	0.25970 (7)	0.35841 (3)	0.0496 (2)	
O1	0.67330 (18)	0.12398 (18)	0.34807 (10)	0.0602 (6)	
O2	0.60372 (18)	0.3428 (2)	0.37858 (10)	0.0666 (6)	
O3	0.94161 (18)	0.2723 (2)	0.27397 (11)	0.0727 (7)	
C1	0.8099 (2)	0.2698 (3)	0.40947 (13)	0.0498 (7)	
C2	0.8117 (3)	0.3717 (3)	0.44912 (14)	0.0652 (9)	
H2A	0.7562	0.4377	0.4476	0.078*	
C3	0.8977 (3)	0.3739 (4)	0.49129 (15)	0.0791 (11)	
H3A	0.8998	0.4410	0.5188	0.095*	
C4	0.9795 (4)	0.2774 (4)	0.49242 (17)	0.0835 (12)	
H4A	1.0372	0.2799	0.5208	0.100*	
C5	0.9782 (3)	0.1777 (4)	0.45296 (17)	0.0797 (11)	
H5A	1.0354	0.1135	0.4542	0.096*	
C6	0.8919 (3)	0.1715 (3)	0.41090 (14)	0.0636 (8)	
H6A	0.8894	0.1026	0.3842	0.076*	
C7	0.7484 (3)	0.3314 (3)	0.29256 (12)	0.0513 (7)	
H7A	0.6841	0.3433	0.2658	0.062*	
H7B	0.7799	0.4174	0.3013	0.062*	
C8	0.8414 (2)	0.2493 (3)	0.26254 (12)	0.0503 (7)	
C9	0.8077 (2)	0.1467 (3)	0.22005 (12)	0.0459 (6)	
C10	0.6939 (2)	0.1183 (3)	0.20632 (13)	0.0520 (7)	
H10A	0.6344	0.1646	0.2243	0.062*	
C11	0.6681 (3)	0.0219 (3)	0.16615 (13)	0.0575 (8)	
H11A	0.5910	0.0031	0.1580	0.069*	
C12	0.7533 (3)	−0.0470 (3)	0.13781 (13)	0.0559 (7)	
C13	0.8667 (3)	−0.0200 (3)	0.15236 (14)	0.0576 (8)	
H13A	0.9258	−0.0669	0.1344	0.069*	
C14	0.8942 (2)	0.0743 (3)	0.19262 (13)	0.0549 (7)	
H14A	0.9714	0.0902	0.2017	0.066*	
C15	0.7230 (3)	−0.1491 (3)	0.09252 (15)	0.0753 (10)	
H15B	0.6483	−0.1854	0.1012	0.113*	
H15C	0.7799	−0.2176	0.0931	0.113*	
H15A	0.7214	−0.1093	0.0545	0.113*	

### Atomic displacement parameters (Å^2^)

**Table:**

	*U*^11^	*U*^22^	*U*^33^	*U*^12^	*U*^13^	*U*^23^
S1	0.0458 (4)	0.0490 (4)	0.0540 (4)	−0.0012 (3)	0.0010 (3)	−0.0013 (3)
O1	0.0659 (14)	0.0448 (12)	0.0700 (14)	−0.0143 (9)	−0.0041 (11)	−0.0014 (10)
O2	0.0541 (13)	0.0746 (16)	0.0710 (15)	0.0124 (10)	0.0071 (11)	−0.0079 (11)
O3	0.0480 (13)	0.0813 (17)	0.0887 (17)	−0.0041 (11)	−0.0105 (12)	−0.0201 (13)
C1	0.0506 (17)	0.0480 (17)	0.0509 (15)	−0.0049 (13)	0.0002 (13)	0.0035 (12)
C2	0.078 (2)	0.057 (2)	0.0599 (19)	−0.0065 (16)	−0.0027 (18)	−0.0040 (15)
C3	0.093 (3)	0.084 (3)	0.061 (2)	−0.024 (2)	−0.007 (2)	−0.0066 (19)
C4	0.074 (3)	0.114 (4)	0.062 (2)	−0.018 (2)	−0.0189 (19)	0.023 (2)
C5	0.067 (2)	0.094 (3)	0.078 (3)	0.006 (2)	−0.007 (2)	0.018 (2)
C6	0.0573 (19)	0.065 (2)	0.068 (2)	0.0057 (15)	−0.0047 (17)	0.0045 (16)
C7	0.0611 (18)	0.0432 (17)	0.0495 (16)	0.0030 (13)	−0.0005 (14)	0.0020 (12)
C8	0.0482 (17)	0.0517 (17)	0.0508 (16)	0.0008 (13)	−0.0023 (13)	0.0039 (13)
C9	0.0421 (15)	0.0466 (16)	0.0491 (15)	0.0022 (11)	0.0002 (13)	0.0034 (12)
C10	0.0408 (15)	0.0613 (18)	0.0540 (17)	0.0053 (13)	0.0030 (14)	0.0020 (13)
C11	0.0465 (18)	0.066 (2)	0.0599 (18)	−0.0071 (14)	−0.0064 (15)	0.0013 (15)
C12	0.0628 (19)	0.0579 (18)	0.0471 (15)	−0.0022 (14)	−0.0031 (16)	0.0031 (14)
C13	0.0544 (19)	0.0594 (19)	0.0590 (18)	0.0086 (13)	0.0040 (16)	−0.0045 (15)
C14	0.0412 (16)	0.0636 (19)	0.0599 (18)	0.0035 (13)	−0.0013 (14)	−0.0034 (14)
C15	0.093 (3)	0.071 (2)	0.061 (2)	−0.0040 (18)	−0.0110 (19)	−0.0085 (17)

### Geometric parameters (Å, º)

**Table:**

S1—O1	1.4287 (19)	C7—H7A	0.9700
S1—O2	1.439 (2)	C7—H7B	0.9700
S1—C1	1.760 (3)	C8—C9	1.480 (4)
S1—C7	1.778 (3)	C9—C10	1.382 (4)
O3—C8	1.210 (3)	C9—C14	1.391 (4)
C1—C2	1.379 (4)	C10—C11	1.377 (4)
C1—C6	1.380 (4)	C10—H10A	0.9300
C2—C3	1.385 (4)	C11—C12	1.373 (4)
C2—H2A	0.9300	C11—H11A	0.9300
C3—C4	1.364 (5)	C12—C13	1.379 (4)
C3—H3A	0.9300	C12—C15	1.509 (4)
C4—C5	1.359 (5)	C13—C14	1.368 (4)
C4—H4A	0.9300	C13—H13A	0.9300
C5—C6	1.386 (5)	C14—H14A	0.9300
C5—H5A	0.9300	C15—H15B	0.9600
C6—H6A	0.9300	C15—H15C	0.9600
C7—C8	1.525 (4)	C15—H15A	0.9600
			
O1—S1—O2	119.10 (13)	H7A—C7—H7B	107.7
O1—S1—C1	107.69 (13)	O3—C8—C9	122.0 (3)
O2—S1—C1	107.92 (14)	O3—C8—C7	118.1 (3)
O1—S1—C7	108.74 (14)	C9—C8—C7	119.9 (2)
O2—S1—C7	106.32 (14)	C10—C9—C14	118.0 (3)
C1—S1—C7	106.42 (14)	C10—C9—C8	123.2 (3)
C2—C1—C6	121.4 (3)	C14—C9—C8	118.8 (3)
C2—C1—S1	119.4 (2)	C11—C10—C9	120.4 (3)
C6—C1—S1	119.1 (2)	C11—C10—H10A	119.8
C1—C2—C3	118.7 (3)	C9—C10—H10A	119.8
C1—C2—H2A	120.7	C12—C11—C10	121.6 (3)
C3—C2—H2A	120.7	C12—C11—H11A	119.2
C4—C3—C2	119.9 (4)	C10—C11—H11A	119.2
C4—C3—H3A	120.0	C11—C12—C13	117.7 (3)
C2—C3—H3A	120.0	C11—C12—C15	120.7 (3)
C5—C4—C3	121.3 (4)	C13—C12—C15	121.6 (3)
C5—C4—H4A	119.3	C14—C13—C12	121.5 (3)
C3—C4—H4A	119.3	C14—C13—H13A	119.2
C4—C5—C6	120.1 (4)	C12—C13—H13A	119.2
C4—C5—H5A	120.0	C13—C14—C9	120.6 (3)
C6—C5—H5A	120.0	C13—C14—H14A	119.7
C1—C6—C5	118.5 (3)	C9—C14—H14A	119.7
C1—C6—H6A	120.7	C12—C15—H15B	109.5
C5—C6—H6A	120.7	C12—C15—H15C	109.5
C8—C7—S1	113.21 (19)	H15B—C15—H15C	109.5
C8—C7—H7A	108.9	C12—C15—H15A	109.5
S1—C7—H7A	108.9	H15B—C15—H15A	109.5
C8—C7—H7B	108.9	H15C—C15—H15A	109.5
S1—C7—H7B	108.9		
			
O1—S1—C1—C2	−146.6 (2)	S1—C7—C8—O3	−92.6 (3)
O2—S1—C1—C2	−16.8 (3)	S1—C7—C8—C9	88.4 (3)
C7—S1—C1—C2	97.0 (3)	O3—C8—C9—C10	179.8 (3)
O1—S1—C1—C6	30.0 (3)	C7—C8—C9—C10	−1.2 (4)
O2—S1—C1—C6	159.8 (2)	O3—C8—C9—C14	0.2 (4)
C7—S1—C1—C6	−86.4 (3)	C7—C8—C9—C14	179.1 (3)
C6—C1—C2—C3	−0.4 (5)	C14—C9—C10—C11	−0.6 (4)
S1—C1—C2—C3	176.1 (2)	C8—C9—C10—C11	179.7 (3)
C1—C2—C3—C4	1.0 (5)	C9—C10—C11—C12	−1.2 (5)
C2—C3—C4—C5	−0.4 (6)	C10—C11—C12—C13	2.3 (5)
C3—C4—C5—C6	−0.8 (6)	C10—C11—C12—C15	−178.0 (3)
C2—C1—C6—C5	−0.8 (5)	C11—C12—C13—C14	−1.5 (5)
S1—C1—C6—C5	−177.3 (3)	C15—C12—C13—C14	178.7 (3)
C4—C5—C6—C1	1.4 (5)	C12—C13—C14—C9	−0.3 (5)
O1—S1—C7—C8	−46.2 (3)	C10—C9—C14—C13	1.4 (4)
O2—S1—C7—C8	−175.5 (2)	C8—C9—C14—C13	−178.9 (3)
C1—S1—C7—C8	69.6 (2)		

### Hydrogen-bond geometry (Å, º)

**Table:**

*D*—H···*A*	*D*—H	H···*A*	*D*···*A*	*D*—H···*A*
C7—H7*B*···O1^i^	0.97	2.42	3.366 (3)	164
C10—H10*A*···O3^ii^	0.93	2.48	3.342 (4)	154

Symmetry codes: (i) −*x*+3/2, *y*+1/2, *z*; (ii) *x*−1/2, *y*, −*z*+1/2.
